# Acoustic Plasmons in Nickel and Its Modification upon Hydrogen Uptake

**DOI:** 10.3390/nano13010141

**Published:** 2022-12-28

**Authors:** Yury M. Koroteev, Igor V. Silkin, Ivan P. Chernov, Evgueni V. Chulkov, Vyacheslav M. Silkin

**Affiliations:** 1 Institute of Strength Physics and Materials Science, Siberian Branch, Russian Academy of Sciences, 634050 Tomsk, Russia; 2 Faculty of Physics, Tomsk State University, Lenin Ave. 36, 634050 Tomsk, Russia; 3 Engineering School of Nuclear Technology, Tomsk Polytechnical University, Lenin Ave. 30, 634050 Tomsk, Russia; 4 Laboratory of Electronic and Spin Structure of Nanosystems, St. Petersburg State University, 198504 St. Petersburg, Russia; 5 Departamento de Polímeros y Materiales Avanzados: Física, Química y Tecnología, Facultad de Ciencias Químicas, Universidad del País Vasco (UPV-EHU), Apdo. 1072, E-20080 San Sebastián, Spain; 6 Donostia International Physics Center (DIPC), Paseo de Manuel Lardizabal 4, E-20018 San Sebastián, Spain; 7 Centro de Fisica de Materiales, Centro Mixto CSIC-UPV/EHU, P. de Manuel Lardizabal, 5, E-20018 San Sebastián, Spain; 8 IKERBASQUE, Basque Foundation for Science, E-48011 Bilbao, Spain

**Keywords:** nickel, hydrides, electronic excitations, plasmons

## Abstract

In this work, we study, in the framework of the ab initio linear-response time-dependent density functional theory, the low-energy collective electronic excitations with characteristic sound-like dispersion, called acoustic plasmons, in bulk ferromagnetic nickel. Since the respective spatial oscillations in slow and fast charge systems involve states with different spins, excitation of such plasmons in nickel should result in the spatial variations in the spin structure as well. We extend our study to NiH${}_{x}$ with different hydrogen concentrations *x*. We vary the hydrogen concentration and trace variations in the acoustic plasmons properties. Finally, at x=1 the acoustic modes disappear in paramagnetic NiH. The explanation of such evolution is based on the changes in the population of different energy bands with hydrogen content variation.

## 1. Introduction

In many metallic systems, the Fermi surface is formed by several energy bands. If the Fermi velocities in these bands are different there is a possibility that a well-defined collective excitation with characteristic sound-line dispersion [1,2] appears in the momentum-energy phase space dominated by the intra-band electron-hole pairs. This excitation termed acoustic plasmon (AP) is a result of the incomplete dynamical screening of the slow carriers by the fast ones [2]. This plasmon can be realized in dilute electron systems in semiconducting heterostructures [3]. For a long time, such kinds of collective electronic excitations in metals and its implication in physical phenomena, such as superconductivity, were discussed [4,5,6,7,8,9,10,11]. Recently, detailed calculations based on the first-principles band structure predicted its existence in some bulk metals such as Pd [12,13], Pb [14], and others [15,16,17,18,19,20]. Low-energy modes with a similar acoustic dispersion can be realized in low-dimensional systems [21,22] and on crystal surfaces [23,24], which support partly occupied electronic surface states as well. They were detected in electron-energy loss spectroscopy [25,26,27,28,29,30] and inelastic atom scattering [31,32] experiments.

Very recently, in calculations of the electronic excitation spectra in bulk ferromagnetic nickel, it was found that a plasmon with a similar sound-like dispersion may exist [31]. In this paper, we extend our previous study of low-energy electronic excitations in nickel and find several acoustic branches with strongly anisotropic dispersion. In particular, we demonstrate that, since the slow and fast electron subsystems in Ni involve carriers with different spins, the resulting plasmonic wave can generate, in addition to the charge imbalance, variations in the spin structure as well. This is because the charge density fluctuations related to the AP excitation correspond to out-of-phase oscillations in the slow and fast valence-charge components [33]. Therefore, we call this kind of modes a spin acoustic plasmon (SAP).

A slope of the AP dispersion, or its group and phase velocities, is mainly determined by the Fermi velocity of the slow component [34], which is several orders of magnitude slower than the speed of light. Therefore, the decay of the surface plasmon resonance, the dynamics of excited electrons and holes [35], or heating in plasmonic materials can be influenced by AP. Taking into consideration the existence of AP might help advance in the understanding of, e.g., the intrinsic photonic efficiency of various photocatalyst materials [36,37,38,39,40] based on Ni.

The AP dispersion can be altered introducing modification to the energy bands that cross the Fermi level, $E_{F}$. One of the ways to reach this consists of absorption of hydrogen resulting in formation of metal hydrides. The metal hydrides have long been studied and still continue to attract much attention due to practical issues such as hydrogen storage and cell fuels as well as fundamental science such as high-temperature superconductivity [41,42,43]. The evolution of AP and its disappearance with the increase of the H concentration was demonstrated in the case of PdH${}_{x}$ [13]. In PdH${}_{x}$ at the H concentrations exceeding x≈0.7 the *d* band becomes totally occupied and the Fermi surface is entirely formed by the s−p electronic states. As a result, the number of energy bands crossing the Fermi level reduced to one. Hence a two-component scenario needed for the AP appearance cannot be realized. In the case of nickel, the uptake of hydrogen in a wide range of concentration can be achieved as well [44]. Moreover, at a high H concentration nickel become paramagnetic. Thus, it would be interesting to trace how the properties of SAPs are modified in NiH${}_{x}$ with the hydrogen concentration.

In this paper, we realize systematic study of the SAP dispersion in Ni finding strong anisotropic behavior in the number of such modes and the dispersing slopes. Upon variation of the absorbed hydrogen content in NiH${}_{x}$ in the zero to unity range of *x*, the evolution of the SAP dispersion and lifetime were traced. In particular, the SAP disappearance in NiH${}_{x}$ at highest *x*’s was found.

The rest of the paper is organized as follows. In Section 2, the details of the *ab initio* computation of the band structure and the dielectric function at small momentum transfers are described. The calculated results are discussed in Section 3. The main results are summarized in Section 4 along with the concluding remarks. Unless otherwise stated, atomic units (*ℏ* = *e* = $m_{e}$ = 1) are used throughout.

## 2. Calculation Methods and Computational Details

The band structure calculations were performed within density functional theory using the full-potential linearized augmented plane wave method (FLAPW) [45], implemented in the FLEUR program package [46]. To describe the exchange-correlation energy, we used the generalized gradient approximation in the Perdew-Burke-Ernzerhof form [47]. The core states were treated fully relativistically, while the valence states were calculated in the scalar-relativistic approximation. The radii of the Ni and H muffin-tin spheres were set to 2.29 and 1.0 a.u., respectively. The value of the cutoff parameter of the plane wave basis kmax was fixed to be of 3.7 a.u. The Brillouin zone (BZ) was sampled over a 12 × 12 × 12 k-point grid. The face-centered cubic (fcc) lattice is used for all H concentrations. At fractional H concentrations the simple cubic lattice unit cell containing four Ni atoms at fcc positions with respective number of H atoms occupying the octahedral sites was used. Bulk lattice constant optimization for each crystal structure was carried out by finding the total energy minimum as a function of the lattice parameter *a*.

The excitation spectra were evaluated in the framework of time-dependent density functional theory [48,49]. The collective electronic excitation—plasmon—characterized by momentum transfer q and energy ω can be identified as a well-defined peak in the loss function, −Im$\left[\epsilon^{-1}\left(q,\omega \right)\right]$. In a periodic bulk crystal, the dielectric function ϵ is defined as a matrix in the reciprocal space. For its expansion we employ the plane waves determined by reciprocal lattice vectors G. The inverse dielectric matrix $\epsilon_{GG^{\prime }}^{-1}\left(q,\omega \right)$ is related to the density response function of interacting electrons $\chi_{GG^{\prime }}\left(q,\omega \right)$ according to

(1) $$\epsilon_{GG^{\prime }}^{-1}\left(q,\omega \right)=\delta_{GG^{\prime }}+\chi_{GG^{\prime }}\left(q,\omega \right)V_{G^{\prime }}\left(q\right),$$

where $\delta_{GG^{\prime }}$ is the unity matrix and $V_{G^{\prime }}\left(q\right)=4\pi /{\left|q+G^{\prime }\right|}^{2}$ the Fourier transform of the bare Coulomb potential. Here we assume that vector q is in the first BZ. The matrix $\chi_{GG^{\prime }}\left(q,\omega \right)$ is obtained from the matrix equation

(2) $$\chi_{GG^{\prime }}\left(q,\omega \right)=\chi_{GG^{\prime }}^{o}\left(q,\omega \right)+\sum_{G_{1},G_{2}}\chi_{GG_{1}}^{o}\left(q,\omega \right)\left[V_{G_{1}}\delta_{G_{1}G_{2}}+K_{G_{1}G_{2}}^{\mathrm{xc}}\left(q,\omega \right)\right]\chi_{G_{2}G^{\prime }}\left(q,\omega \right).$$

The kernel $K_{\mathrm{xc}}$ accounts for the exchange-correlation effects. In the present work we employ the random-phase approximation (RPA) where $K_{\mathrm{xc}}$ is set to zero, since, in general, the impact of these effects beyond the RPA at q’s of interest here is small [50]. In Equation (2) $\chi_{GG^{\prime }}^{o}\left(q,\omega \right)$ is the response function of the non-interacting Kohn-Sham electrons that is defined as

(3) $$\begin{array}{ccc} \chi_{GG^{\prime }}^{o}\left(q,\omega \right) & = & \frac{1}{\Omega }\sum_{k,s}^{\mathrm{BZ}}\sum_{n}^{\mathrm{occ}}\sum_{n^{\prime }}^{\mathrm{unocc}}\frac{f_{nsk}-f_{n^{\prime }sk+q}}{\varepsilon_{nsk}-\varepsilon_{n^{\prime }sk+q}+\left(\omega +i\eta \right)}\\ & \times & \langle \psi_{nsk}|e^{-i(q+G)r}|\psi_{n^{\prime }sk+q}\rangle \langle \psi_{n^{\prime }sk+q}|e^{i(q+G^{\prime })r}|\psi_{nsk}\rangle , \end{array}$$

where Ω is the unit cell volume, $f_{nsk}$ is the Fermi occupation number at zero temperature, η infinitesimal, and summation over spin is explicitly taken into account. Summation over the BZ in systems containing four Ni atoms in a unit cell is realized on a 200×200×200 grid. For fcc Ni and NiH the meshes with equivalent spacing between k points were employed. Since we are interested in electronic excitations in the energy region dominated by intra-band transitions, only such transitions with probability set to unity were included in Equation (3). As a consequence, the energy bands crossing the Fermi level were taken into account in evaluation of $\chi^{o}$. We expect that inclusion of interband transitions do not introduce notable effect on the modes found here since there is some energy threshold for such transitions. On the other hand, variations in the real part of dielectric function produced by such transitions indirectly in the considered energy interval are in general rather small in comparison to the effect caused by intraband transitions [50]. In the expansion of matrixes $\chi^{o}$, χ, and $\epsilon^{-1}$ a single G = 0 vector was included. Further calculations details can be found elsewhere [51].

## 3. Calculation Results and Discussion

### 3.1. A Bare Nickel

**Figure 1:**
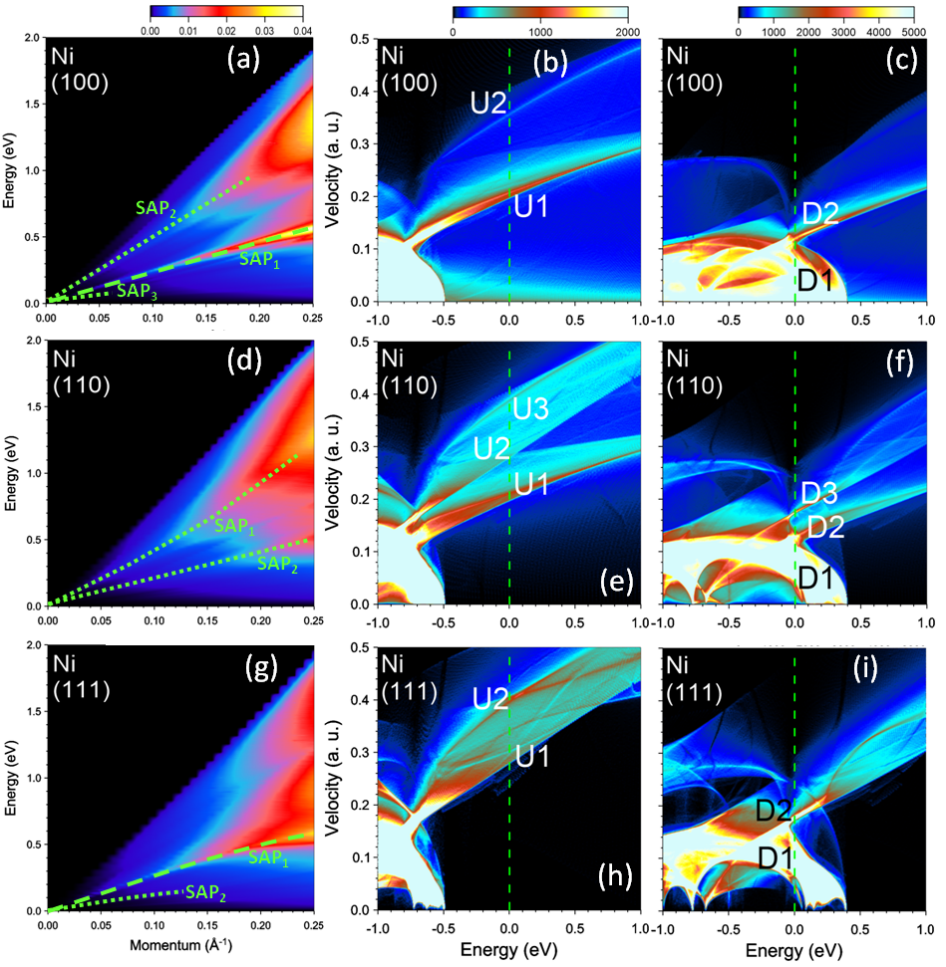
Loss function in bare nickel at the momentum transfers along (**a**) [100], (**d**) [110], and (**g**) [111] symmetry directions. The well-defined (weak) peaks are highlighted by green dashed (dotted) lines. Density of states (DOS) versus energy and group velocity component along the respective directions for the (**b**,**e**,**h**) majority and (**c**,**f**,**i**) minority spins. Most prominent peaks at the Fermi level (vertical green dashed line) in the DOS are marked by symbols.

We start by presenting the calculation results for clean nickel. In Figure 1a, we show the imaginary part of the inverse dielectric function, −Im$\left[\epsilon^{-1}\left(q,\omega \right)\right]$, as a function of the q value and energy ω. The vectors q are along [100] symmetry direction. Here one can observe several peaks. In the low-energy part, the excitation spectrum is dominated by a sharp peak SAP${}_{1}$ with a clear sound-like dispersion, i.e., its energy tends to zero upon momentum reduction, and the dispersion has almost linear dependence on *q*. To reveal its origin, it is helpful to analyze the shape of the density of states (DOS) as a function of energy *E* and group velocity υ in the Fermi level vicinity [12]. In Figure 1b,c, we report the DOS$\left(E,\upsilon_{100}\right)$ versus *E* and $\upsilon_{100}$, where $\upsilon_{100}$ denotes a group velocity component along [100] symmetry direction. In the majority-spin DOS (Figure 1b), besides a featureless background, one can see a sharp peak U1 with the Fermi velocity value of about 0.2 a.u. Another weak peak marked as U2 can be discerned at upper energies. Its Fermi level value is of 0.355 a.u. All the states in the Fermi level vicinity are of the *s*-*p* character, since the *d*-like states with majority-spin reside at energies below −0.5 eV. In the case of states with the minority spin the respective DOS in Figure 1c at the Fermi level is dominated by the low velocity *d* states. In the DOS at $E_{F}$ these states generate a broad prominent peak D1 with the velocity values below 0.1 a.u. A second narrow peak D2 has a value of 0.12 a.u. at $E_{F}$.

**Figure 2:**
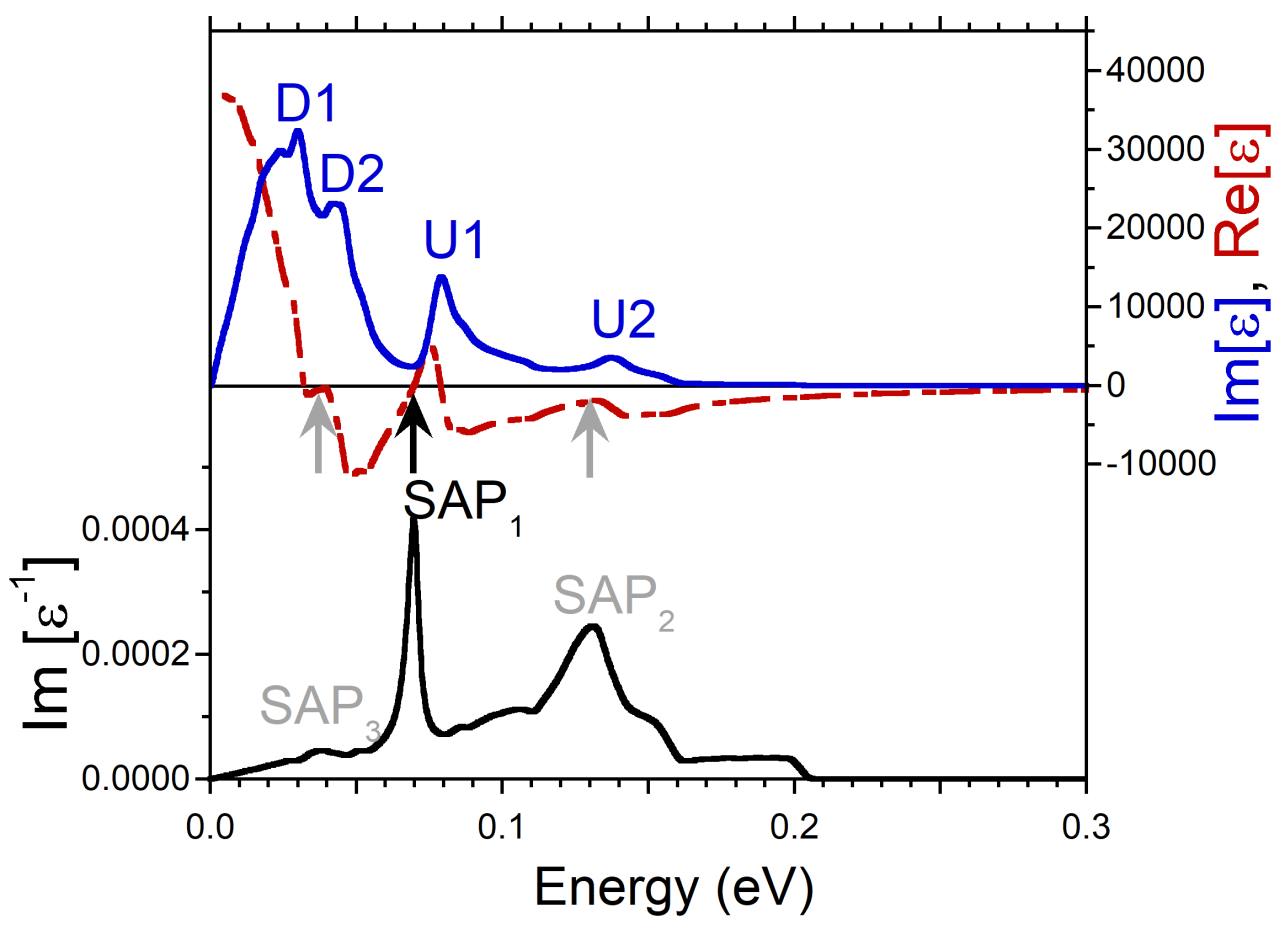
Real (red dashed line) and imaginary (blue solid line) parts of dielectric function in Ni calculated at *q* = 0.027 Å${}^{-1}$ pointing in the [100] symmetry direction and respective loss function, −Im$\left[\epsilon^{-1}\right]$, (black solid line). Four peaks in Im[ϵ] generated by the states marked in the DOS of Figure 1b,c are labeled by the respective symbols. The energy region where Re[ϵ] crosses with positive slope the zero line is marked by black arrow, whereas the regions where it approaches the zero line only are marked by gray arrows. In the loss function, a peak corresponding to a long-lived spin acoustic plasmon is marked as SAP${}_{1}$. The strongly damped acoustic plasmon modes are denoted as SAP${}_{2}$ and SAP${}_{3}$.

At small q’s, the energy positions of the peaks in Im[ϵ] produced by the intra-band transitions can be determined rather well as a product of *q* and the Fermi velocity of the peaks in the DOS. It is exactly what one can observe in the imaginary part of dielectric function reported in Figure 2. The calculations were realized at q=0.027 Å${}^{-1}$. There is a prominent peak D1 related to the minority spin peak D1 in the DOS of Figure 1c. Next peak D2 in Im[ϵ] of Figure 2 is connected to the peak D2 in the DOS of Figure 1c. At the higher energies, in Im[ϵ] of Figure 2 the other two peaks U1 and U2 are produced by the faster moving majority-spin carriers with the Fermi velocities determined by the peaks U1 and U2 in Figure 1b, respectively. Notice that both the energy positions of the peaks in Im[ϵ] and the relative strength of these peaks are proportional to the Fermi velocity and the amplitude of the peaks in the DOS. Therefore, for instance, a weak and diffuse peak U2 in the DOS of Figure 1b generates a relatively weak and broad peak U2 in Im[ϵ] of Figure 2.

Since the real and imaginary parts of the complex dielectric function are connected via the Kramers-Kronig relation, such a multi-peak structure of the intra-band part of Im[ϵ] is reflected in Re[ϵ] as well. Indeed, it is seen in Figure 2 that Re[ϵ] has strongly varying behavior in this energy range, in contrast to what is expected from a free-electron gas model [52,53]. In particular, as marked by the black arrow, it crosses the zero line with positive slope at energy around 0.07 eV. In combination with a presence of a local minimum in Im[ϵ] in the nearby energy region this zero-crossing produces a well-defined peak in the loss function at a close energy. Since the nearest-energy peaks D2 and U1 in Im[ϵ] are related to the states with different spins, one can consider the respective collective excitation as the out-of-phase oscillations of both the charge and spin densities. Therefore, we call this mode the spin acoustic plasmon (SAP) and denote the respective peak in the loss function of Figure 2 as SAP${}_{1}$. Notice that in the case of SAP, the variations in spin structure occur due to charge density oscillations in electron subsystems with different spin orientations in contrast to the case of a magnon, a quasiparticle corresponding to a collective excitation of the electrons’ spin structure only [54].

Additionally, in the loss function of Figure 2, a wide peak marked as SAP${}_{2}$ centered at energy of 0.13 eV is seen. In contrast to SAP${}_{1}$, we do not find a zero-crossing in Re[ϵ] in the nearby energy region. Instead, Re[ϵ] only approaches zero as highlighted by gray arrow. However, since the peak SAP${}_{2}$ emerges in the loss function at energies where Im[ϵ] has a shallow local minimum, the respective excitation can be classified as a strongly damped plasmon. The respective charge oscillations involve the majority-spin states only. In the loss function of Figure 2, on the low-energy side, one can also discern a weak peak SAP${}_{3}$ at energy of 0.037 eV which we interpret as a weak acoustic plasmon as well. We relate its existence to the fact that Re[ϵ] approaches zero (as marked by gray arrow) and Im[ϵ] has a local minimum in this energy interval. The weakness of this mode can be explained by relatively small depth of the respective minimum in Im[ϵ]. Since the SAP${}_{3}$ peak in Im$\left[\epsilon^{-1}\right]$ occurs in the energy window between the peaks D1 and D2, this weak mode corresponds to the out-of-phase charge oscillations in the minority spin channel.

**Figure 3:**
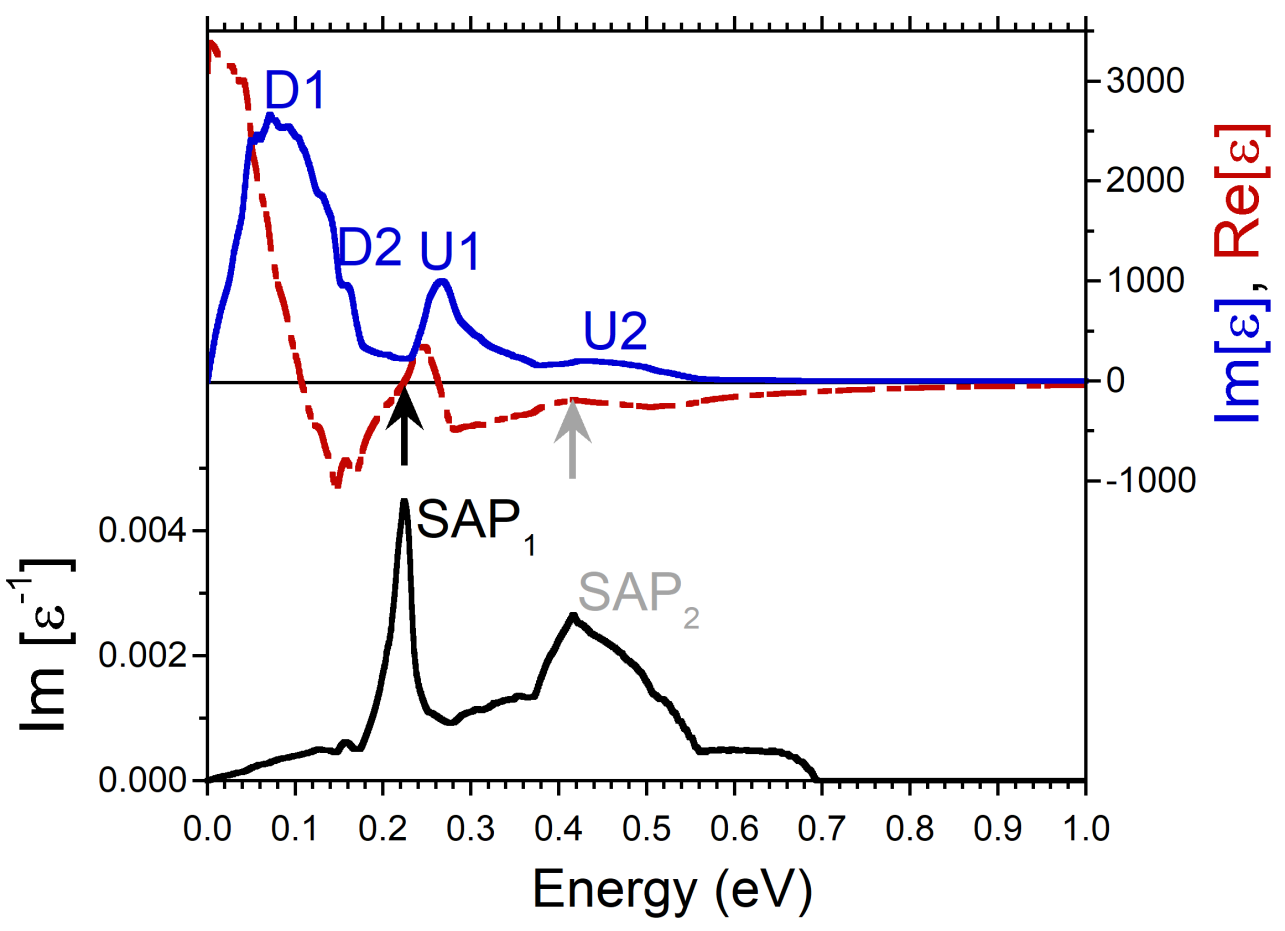
Real (red dashed line) and imaginary (blue solid line) parts of dielectric function in Ni calculated at *q* = 0.089 Å${}^{-1}$ pointing in [100] symmetry direction and respective loss function, −Im$\left[\epsilon^{-1}\right]$, (black solid line). Four peaks in Im[ϵ] generated by the states marked in the DOS of Figure 1b,c are labeled by the respective symbols. The energy region where Re[ϵ] crosses (approaches) with positive slope the zero line is marked by black (gray) arrow. In the loss function a peak corresponding to a long-lived spin acoustic plasmon is marked as SAP${}_{1}$. The strongly damped acoustic plasmon peak is denoted as SAP${}_{2}$.

On increasing the momentum transfer q, more states beyond the Fermi level vicinity become involved in the formation of dielectric function. For illustration, in Figure 3, we report the dielectric function and the loss function evaluated at momentum transfer with magnitude of 0.089 Å${}^{-1}$ along the same [100] symmetry direction. One can see that the peaks in Im[ϵ] become wider in comparison to Figure 2. As a result, the minority-spin peak D2 almost merges to the dominating peak D1. Furthermore, the peak U2 becomes notably weaker. As a result, the width of the SAP${}_{2}$ peak in the loss function significantly increases, reflecting a reduction of its lifetime. Furthermore, the intensity of the SAP${}_{2}$ peak is notably reduced signalling about losing its collective nature. On the low-energy side of the loss function we do not find any collective mode anymore. A small peak at ω = 0.16 eV is not a collective excitation since its energy position coincides with that of the D2 peak in Im[ϵ]. Hence, this peak in Im$\left[\epsilon^{-1}\right]$ simply corresponds to the enhanced number of the single-particle electron-hole-pair excitations.

**Figure 4:**
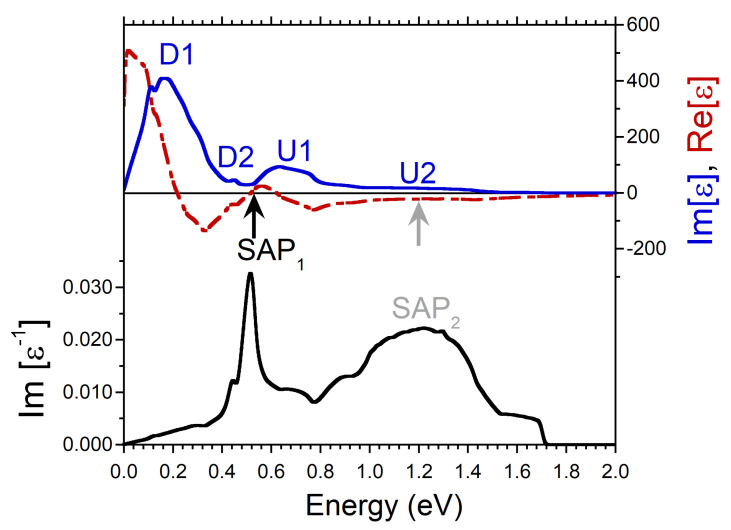
Real (red dashed line) and imaginary (blue solid line) parts of dielectric function in Ni calculated at *q* = 0.223 Å${}^{-1}$ pointing in [100] symmetry direction and the respective loss function, −Im$\left[\epsilon^{-1}\right]$, (black solid line). Four peaks in Im[ϵ] generated by the states marked in the DOS of Figure 1b,c are labeled by the respective symbols. The energy region where Re[ϵ] crosses (approaches) with positive slope the zero line is marked by black (gray) arrow. In the loss function a peak corresponding to a long-lived spin acoustic plasmon is marked as SAP${}_{1}$. The overdamped acoustic plasmon peak is denoted as SAP${}_{2}$.

Once *q* increases up to 0.223 Å${}^{-1}$, ϵ and Im$\left[\epsilon^{-1}\right]$ experience further modifications, as seen in Figure 4. Thus, the D2 peak in Im[ϵ] becomes extremely small, whereas the feature U2 presents almost flat shape. Nevertheless, at this *q* the SAP${}_{1}$ peak is still rather sharp since the two conditions (a zero-crossing in Re[ϵ] as pointed by black arrow and a local minimum in Im[ϵ]) are fulfilled. As a result, the respective collective mode may be well defined. However, we do not expect that this can be realized experimentally since at this energy the inclusion of the numerous inter-band transitions most probably should destroy such a mode. On the other hand, the broad peak SAP${}_{2}$ cannot be considered as a signature of a collective excitation, since the local minimum of Im[ϵ] is hardly visible in the respective energy region whereas in Re[ϵ] the zero approaching (highlighted by gray arrow) cannot be resolved on this scale.

**Figure 5:**
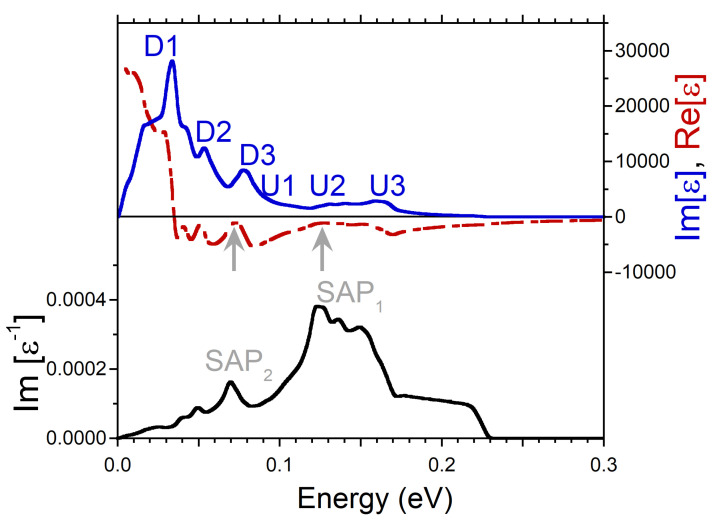
Real (red dashed line) and imaginary (blue solid line) parts of dielectric function in Ni calculated at *q* = 0.032 Å${}^{-1}$ pointing in [110] symmetry direction and respective loss function, −Im$\left[\epsilon^{-1}\right]$, (black solid line). Features in Im[ϵ] generated by the states marked in the DOS of Figure 1e,f are labeled by respective symbols. The energy regions where Re[ϵ] approaches the zero line are marked by gray arrows. Features in the loss function corresponding to strongly damped spin acoustic plasmon modes are denoted as SAP${}_{1}$ and SAP${}_{2}$.

Changing the direction of q significantly affects the excitation spectra in Ni. In Figure 1d, we present the loss function calculated at momentum transfers along the [110] symmetry direction. One can see that the spectra is dominated by two weak peaks denoted as SAP${}_{1}$ and SAP${}_{2}$ with characteristic sound-like dispersion. However, in contrast to the [100] direction, neither of these peaks can be considered a true acoustic plasmon. We relate this to the larger number of the one-particle states with different Fermi velocities presented in the calculated DOS for this symmetry direction. As seen in Figure 1e,f in the DOS the number of such states increases up to three for each spin. Moreover the peaks denoted as U2 and U3 in the DOS of Figure 1e are rather broad and relatively weak. This results in the less pronounced peaks in the imaginary part of dielectric function. This is illustrated in Figure 5 where the dielectric and loss functions calculated at *q* = 0.032 Å${}^{-1}$ along the [110] direction are shown. In Im[ϵ] we identified several features whose origin can be traced to the peaks in the DOS of Figure 1e,f. However, the real part of ϵ does not cross zero with a positive slope in this energy interval. It only approaches this line at some energies as illustrated by gray arrows. The resulting peaks in the loss function marked as SAP${}_{1}$ and SAP${}_{2}$ we interpret as strongly mixed electron-hole-plasmon excitations. These peaks in the loss function can be traced at *q*’s almost up to 0.25 Å${}^{-1}$. However, the width of the peaks (the spectral weight) strongly increases (reduces) with increasing momentum transfers.

**Figure 6:**
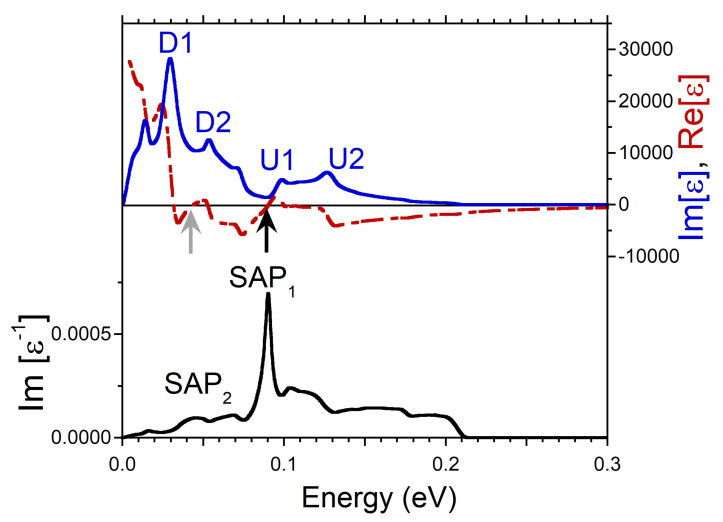
Real (red dashed line) and imaginary (blue solid line) parts of dielectric function in Ni calculated at *q* = 0.038 Å${}^{-1}$ pointing in [111] symmetry direction and respective loss function, −Im$\left[\epsilon^{-1}\right]$, (black solid line). Features in Im[ϵ] generated by the states marked in the DOS of Figure 1h,i are labeled by respective symbols. The energy regions where Re[ϵ] crosses the zero line with positive slope are marked by arrows. In the loss function a peak corresponding to a long-lived spin acoustic plasmon is marked as SAP${}_{1}$. The overdamped acoustic plasmon peak is denoted as SAP${}_{2}$.

In the case of *q*’s directed along the [111] direction the excitation spectrum reported in Figure 1g is dominated by the acoustic plasmon peak marked as SAP${}_{1}$. To understand its origin in Figure 6 we plot the respective dielectric and loss functions evaluated at q=0.038 Å${}^{-1}$. Here, even though the peaks D1, U1, and U2 in Im[ϵ] are rather weak, a pronounced minimum at energies around 0.09 eV and steep increase of the peak U1 at higher energies ensure that Re[ϵ] crosses zero in this region. As a result, a sharp peak SAP${}_{1}$ corresponding to a well-defined acoustic plasmon appears in the loss function. Again, as in the case of SAP${}_{1}$ along [100] direction, this mode corresponds to out-of-phase charge oscillations involving states with different spins, namely, in the minority-spin peak D2 in the DOS shown in Figure 1i and in the majority-spin peak U1 in Figure 1h.

Additionally, on the lower energy side of the loss function of Figure 6, a weak peak marked as SAP${}_{2}$ can be detected at 0.045 eV. Considering that there is a zero-crossing in Re[ϵ] and a local minimum in Im[ϵ] at nearby energies, we interpret this peak as an acoustic plasmon as well. However, the low intensity and rather wide width of the peak signal about small spectral weight of this feature. In the loss function of Figure 1g we can trace this peak up to about 0.15 eV.

It is well known that in the ferromagnetic Ni the low-energy excitations are dominated by spin waves or magnons, whose energy goes to zero when q→0 [55,56,57,58,59,60,61]. Despite the fact that the acoustic plasmon modes discussed in this work also have vanishing energies when the momentum transfer approaches zero, the direct interaction of these modes with magnons is not possible. This is because the magnon dispersion at small *q*’s has quadratic dependence on the momentum, whereas acoustic plasmons possess a linear dispersion. Moreover, we expect that efficient decay into incoherent electron-hole pairs does not allow the existence of the acoustic plasmons over an extended momentum range whereas magnons in Ni can exist over a whole BZ [59,60].

### 3.2. NiH${}_{x}$

**Figure 7:**
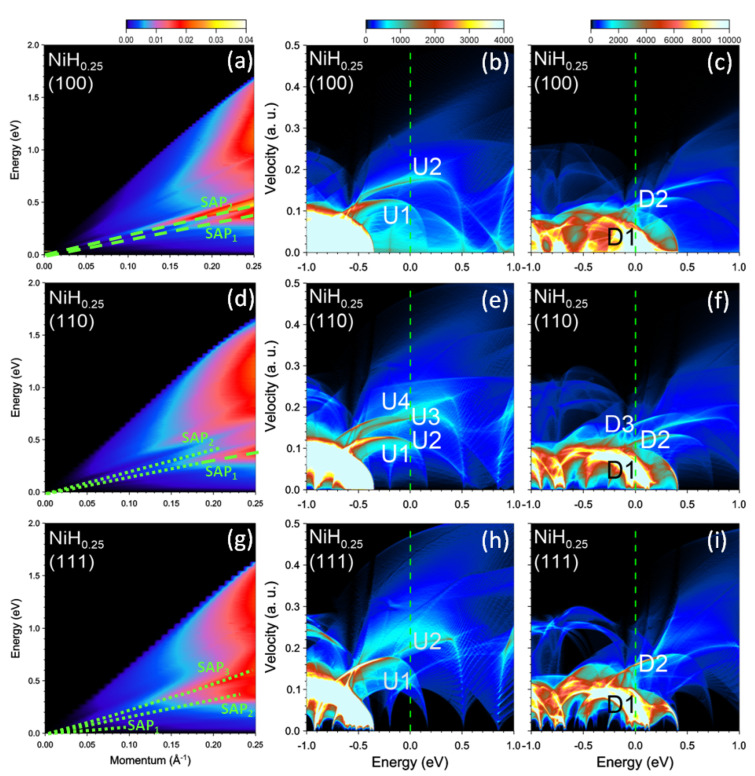
Loss function in NiH${}_{0.25}$ at the momentum transfers along the (**a**) [100], (**d**) [110], and (**g**) [111] symmetry directions. The well-defined (weak) peaks are highlighted by green dashed (dotted) lines. Density of states (DOS) versus energy and group velocity component along the respective directions for the (**b**,**e**,**h**) majority and (**c**,**f**,**i**) minority spins. The most prominent peaks at the Fermi level (vertical green dashed line) in the DOS are marked by symbols.

**Figure 8:**
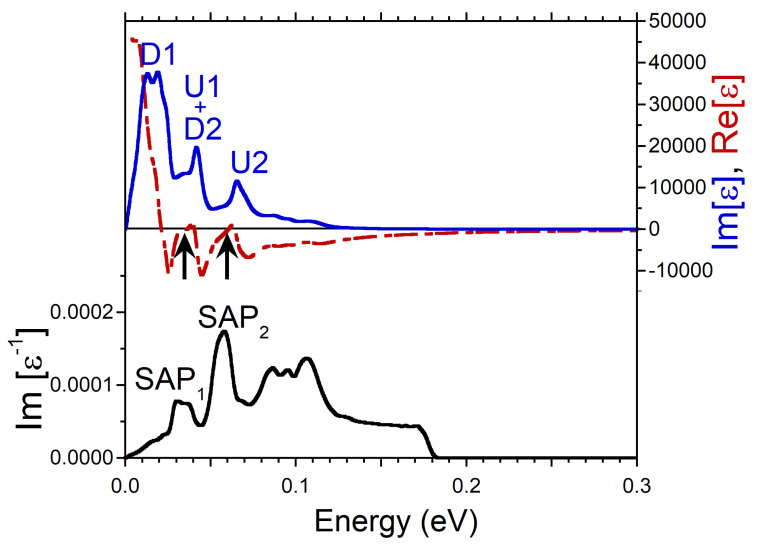
Real (red dashed line) and imaginary (blue solid line) parts of dielectric function in NiH${}_{0.25}$ calculated at *q* = 0.027 Å${}^{-1}$ pointing in [100] symmetry direction and respective loss function, −Im$\left[\epsilon^{-1}\right]$, (black solid line). Three peaks in Im[ϵ] generated by the states marked in the DOS of Figure 7b,c are labeled by respective symbols. The energy regions where Re[ϵ] crosses the zero line with positive slope are marked by arrows. In the loss function a peak corresponding to a long-lived spin acoustic plasmon is marked as SAP${}_{2}$. A weaker acoustic plasmon peak is denoted as SAP${}_{1}$.

Absorption of hydrogen results in notable modifications in the electronic structure of the host nickel [62,63,64]. In particular, the DOS at the Fermi surface in NiH${}_{x}$ depends sensitively on the hydrogen concentration *x*. One of the trends consists of approaching the top of the majority-spin *d* band to the Fermi level upon increase of *x* and downward shift of the minority-spin *d* band which results in the reduction of magnetization. The above shifts can be observed in comparing the DOS in NiH${}_{0.25}$ reported in Figure 7 with that of a pure nickel presented in Figure 1. Thus, the top of the majority-spin *d* band in NiH${}_{0.25}$ locates at −0.35 eV whereas in Ni it is observed at −0.48 eV. In the case of the minority spin *d* band the situation is more involved. One can notice that above the Fermi level this band splits into a separate band with energy of 0.41 eV at zero velocity in any direction (corresponding to the BZ center) and the bands producing the high DOS below 0.30 eV along the [100] direction (Figure 7c) and below 0.14 eV in two other directions (Figure 7f,i). Moreover, one can see that the peak distribution in the DOS at $E_{F}$ changes significantly. In general, the group velocities in all such peaks become substantially smaller with subsequent changing the energy intervals between them. This strongly affects the excitation spectra of NiH${}_{0.25}$. Thus, the loss function in [100] direction of Figure 7a presents now two peaks, SAP${}_{1}$ and SAP${}_{2}$, corresponding to acoustic modes. A comparison of Figure 1a and Figure 7a reveals strong alteration in the dispersion slopes of the SAP${}_{1}$ and SAP${}_{2}$ peaks, whereas the peak SAP${}_{3}$ disappears in NiH${}_{0.25}$. Moreover, the strength of the SAP${}_{2}$ increases, whereas the SAP${}_{1}$ peak becomes broader. For illustration, in Figure 8 we report the dielectric and loss functions in NiH${}_{0.25}$ evaluated at q=0.027 Å${}^{-1}$ along [100] direction. One can see that the peaks in Im[ϵ] lie lower in energy than in Ni in Figure 2. Moreover, the number of the well-defined peaks in Im[ϵ] reduces to three since the Fermi velocities of the peaks U1 and D2 in the DOS of Figure 7b,c become very close. In contrast to Figure 2, the real part of ϵ in Figure 8 crosses clearly the zero line with positive slopes two times. Consequently, the SAP${}_{2}$ in the loss function becomes better defined as a collective excitation. One can understand this mode as the out-of-phase charge (and spin) oscillations involving the states forming the U1, D2, and U2 peaks in the DOS. In contrast, the strength and sharpness of the SAP${}_{1}$ peak are notably suppressed in Figure 8. Nevertheless, based on the criteria for the existence of a collective electronic excitation, we interpret this feature as a true plasmon as well. The respective charge and spin oscillations include mainly the states in the D1, U1, and D2 peaks in the DOS of Figure 7b,c.

**Figure 9:**
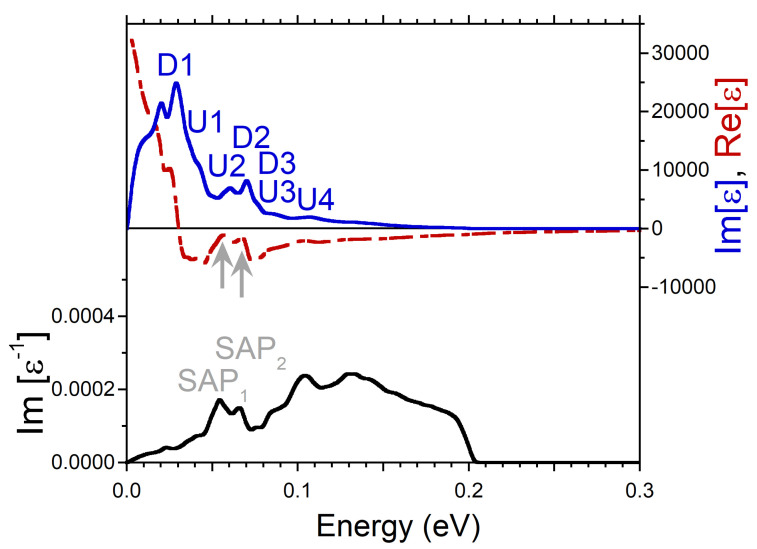
Real (red dashed line) and imaginary (blue solid line) parts of dielectric function in NiH${}_{0.25}$ calculated at *q* = 0.032 Å${}^{-1}$ pointing in [110] symmetry direction and respective loss function, −Im$\left[\epsilon^{-1}\right]$, (black solid line). Features in Im[ϵ] generated by the states marked in the DOS of Figure 7e,f are labeled by respective symbols. The energy regions where Re[ϵ] approaches the zero line are marked by gray arrows. Features in the loss function corresponding to strongly damped acoustic plasmon modes are denoted as SAP${}_{1}$ and SAP${}_{2}$.

In the case of the loss function for the [110] symmetry direction reported in Figure 7d, two weak peaks corresponding to significantly damped acoustic plasmons are highlighted as SAP${}_{1}$ and SAP${}_{2}$. Again, the dispersion slopes of these modes are significantly lower than those of pure nickel. The reduction of energy separation between these two peaks in the loss function can mainly be explained by a larger number of peaks with different Fermi velocities in the DOS as can be deduced from Figure 7e,f. The upper-energy peak SAP${}_{2}$ is rather weak at any *q*. At small *q*’s the peak SAP${}_{1}$ also cannot be considered a well-defined collective excitation. This can be seen from Figure 9 where the dielectric and loss functions evaluated at q=0.032 Å${}^{-1}$ are presented. For instance, in Im[ϵ] the energy separation between the peaks is rather small due to small difference in the Fermi velocities between respective peaks in the DOS of Figure 7e,f. As a consequence, the real part does not reach zero in this energy region and only a weak peak at energy of 0.055 eV emerges in the loss function. Curiously, the spectral weight of this peak increases at larger momentum transfers and it becomes significantly better defined at larger momentum transfer beyond q≈0.2 Å${}^{-1}$.

**Figure 10:**
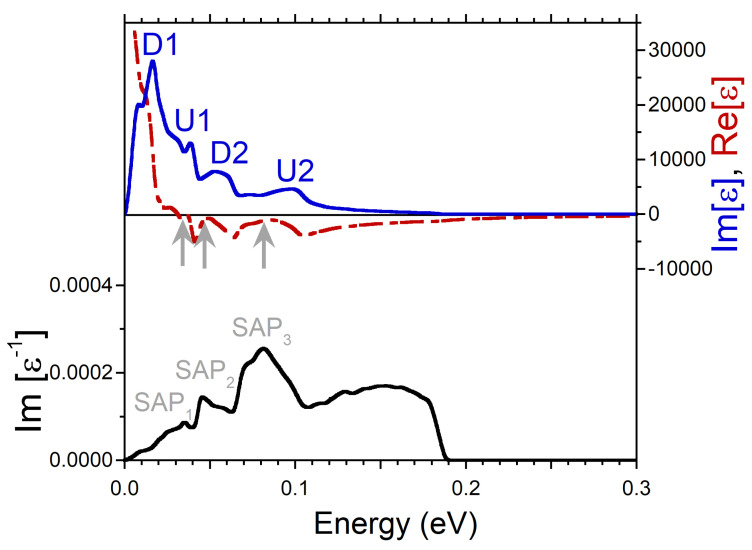
Real (red dashed line) and imaginary (blue solid line) parts of dielectric function in NiH${}_{0.25}$ calculated at *q* = 0.038 Å${}^{-1}$ pointing in [111] symmetry direction and respective loss function, −Im$\left[\epsilon^{-1}\right]$, (black solid line). Features in Im[ϵ] generated by the states marked in the DOS of Figure 7e,f are labeled by respective symbols. The energy regions where Re[ϵ] approaches the zero line are marked by gray arrows. Peaks in the loss function corresponding to strongly damped acoustic plasmon modes are denoted as SAP${}_{1}$, SAP${}_{2}$, and SAP${}_{3}$.

At momentum transfers along the [111] direction the loss function shown in Figure 7g presents three features marked by symbols SAP${}_{1}$, SAP${}_{2}$, and SAP${}_{3}$. We interpret them as strongly damped acoustic plasmons. One can notice that the SAP${}_{3}$ peak dispersion almost coincides with that of the SAP${}_{2}$ peak of Figure 1g in pure nickel. However, in NiH${}_{0.25}$ this peak in the loss function is significantly wider, i.e., its lifetime becomes shorter. Moreover, as seen in Figure 10 the real part of the dielectric function does not reach zero in the nearby energy interval. As for the SAP${}_{1}$ and SAP${}_{2}$ modes, even though Re[ϵ] in Figure 10 approaches closely zero at the respective energies, the peaks in the loss function are rather weak because of the absence of well-defined dips in Im[ϵ].

In general, we can conclude this part noting that the SAPs in NiH${}_{0.25}$ become weaker than in the pure nickel. We relate this observation to the smaller Fermi velocities of the states forming pronounced peaks in the DOS. In particular, this is observed for the majority-spin states. As a result, the energy separation between the peaks and their identificaton in the imaginary part of dielectric function are reduced, which disfavors the realization of acoustic plasmons.

**Figure 11:**
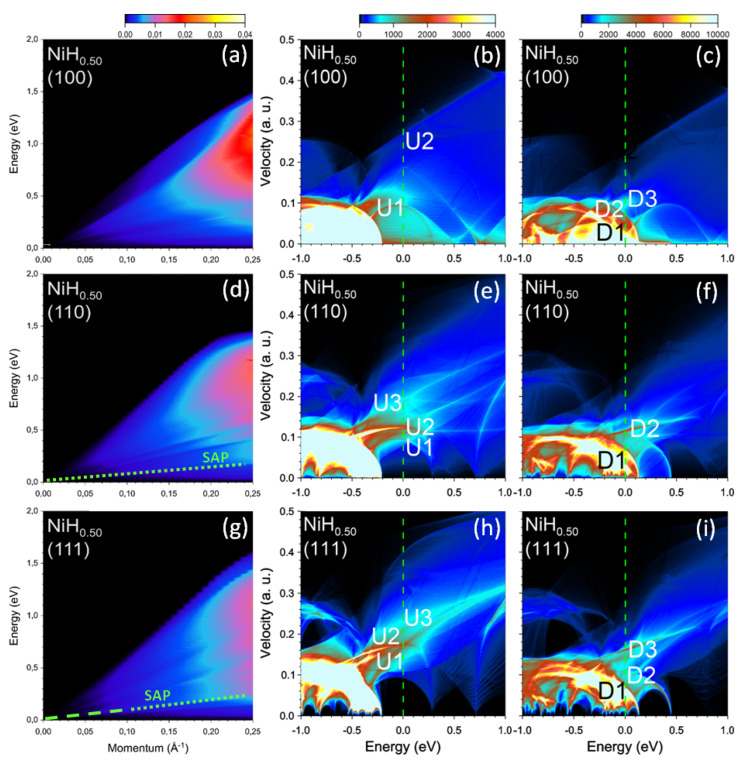
Loss function in NiH${}_{0.50}$ at the momentum transfers along (**a**) [100], (**d**) [110], and (**g**) [111] symmetry directions. The well-defined (weak) peaks are highlighted by green dashed (dotted) lines. Density of states (DOS) versus energy and group velocity component along the respective directions for the (**b**,**e**,**h**) majority and (**c**,**f**,**i**) minority spins. Most prominent peaks at the Fermi level (vertical green dashed line) in the DOS are marked by symbols.

Increasing of hydrogen content up to x=0.50 continues the trend of the reduction of magnetization caused by a smaller splitting between the states with majority and minority spins. This can be seen in the DOS plots reported in Figure 11. In this case the top of the majority-spin *d* band is placed at −0.20 eV, whereas the intense minority spin peak in the DOS is seen below 0.13 eV. Furthermore, the intensity of the separate *d* band with a top at 0.44 eV (where it has zero group velocity) is notably reduced in comparison to NiH${}_{0.25}$. This is accompanied by a redistribution in the peak positions and intensities in the DOS of the states crossing the Fermi level. As seen in the DOS of Figure 11b,c the most intense peaks at the Fermi level for both spins span almost the same energy interval (up to about 0.12 a.u.). Moreover, the intensity of peaks U1, U2, and D3 are very low. This results in the absence of a well-defined separate-peaks structure in the imaginary part of the dielectric function at any q along [100] direction. In turn, the real part becomes rather smooth. All this leads to a featureless behavior of the loss function reported in Figure 11a. All this region is dominated by incoherent electron-hole pairs.

When q points in the [110] direction, the respective loss function reported in Figure 11d becomes essentially featureless as well. The only weak peak marked as SAP can be resolved in the low-energy part. From analysis of the dielectric function, we interpret this feature as a weak SAP corresponding to out-of-phase charge oscillations involving the states that form the U1, U2, and D2 peaks in the DOS reported in Figure 11e,f. Some other weak features can be detected in Figure 11d at upper energies as well. However, analyzing the dielectric function behavior, we interpret these features as single-particle excitations. A similar SAP peak can be detected in Figure 11g where the loss function evaluated at momentum transfers along the [111] direction is presented. However, contrary to the SAP peak in Figure 11d, this peak in the loss function of Figure 11g is rather sharp for *q*’s up to about 0.10 Å${}^{-1}$. We interpret it as a collective excitation. At *q*’s beyond this limit this mode gradually degrades and looses its collective nature.

**Figure 12:**
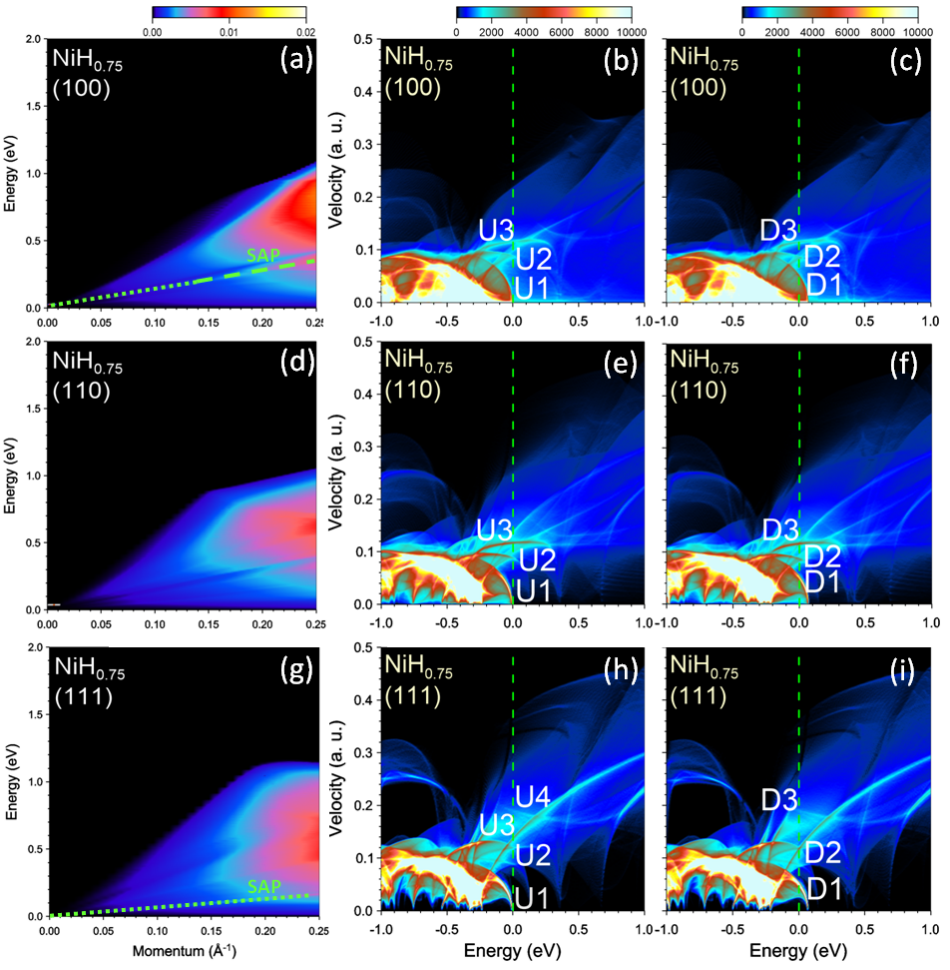
Loss function in NiH${}_{0.75}$ at the momentum transfers along (**a**) [100], (**d**) [110], and (**g**) [111] symmetry directions. The well-defined (weak) peaks are highlighted by green dashed (dotted) lines. Density of states (DOS) versus energy and group velocity component along the respective directions for the (**b**,**e**,**h**) majority and (**c**,**f**,**i**) minority spins. Most prominent peaks at the Fermi level (vertical green dashed line) in the DOS are marked by symbols.

At the hydrogen concentration x=0.75, the energy splitting in our calculations between the states with the majority and minority spins becomes less than 0.1 eV as can be deduced from the DOS reported in central and right columns of Figure 12. This results in a very small difference in the Fermi velocities of the peaks in the DOS between the two subsystems with different spins. Therefore the separate-peaks structure in Im[ϵ] comes mainly from the differences in the Fermi velocities of the DOS peaks in each spin subsystem. The only exceptions are the U1 and D1 bands due to its strong dispersion around the BZ center. Thus, the top of the U1 band only touches the Fermi level with almost zero velocity, whereas in the D1 band the Fermi velocity is about 0.05 a.u. The smooth variation of Im[ϵ] results in a rather featureless behavior of the loss function reported in the left column of Figure 12. Apart from this, in the loss function of Figure 12a at momentum transfers larger than ≈0.14 Å${}^{-1}$ we detect a peak corresponding to a collective excitation. A peak with significantly smaller spectral weight corresponding to a collective excitation can be detected at any *q* along [111] direction in the loss function reported in Figure 12g.

**Figure 13:**
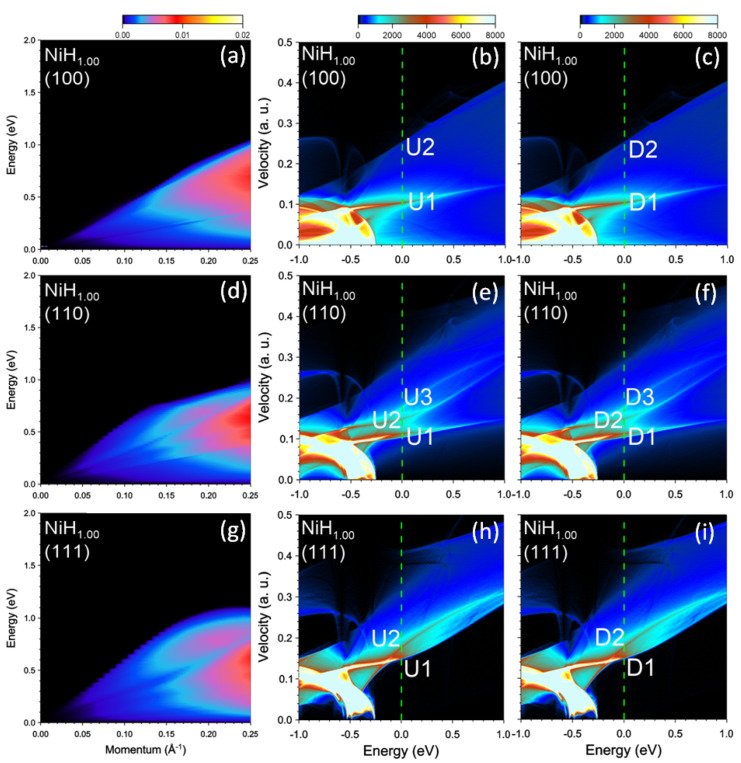
Loss function in NiH${}_{1.00}$ at the momentum transfers along (**a**) [100], (**d**) [110], and (**g**) [111] symmetry directions. Density of states (DOS) versus energy and group velocity component along the respective directions for the (**b**,**e**,**h**) majority and (**c**,**f**,**i**) minority spins. Most prominent peaks at the Fermi level (vertical green dashed line) in the DOS are marked by symbols.

In NiH, the electron doping due to hydrogen results in significant downward shift of the *d* bands below the Fermi level. In the DOS presented in the central and right columns of Figure 13 one can see that now the top of *d* bands is at −0.25 eV and the Fermi surface is formed by the states with s−p character. Furthermore, the spin splitting between the bands with different spins vanishes (notice the equivalence of the DOS in the central and right columns). Nevertheless, one can resolve two peaks (the U2 and D2 peaks are hardly seen in Figure 13b,c) with different Fermi velocities in the DOS for the [100] and [111] directions. In the case of the DOS reported in Figure 13e,f we resolve three such groups. However, despite the presence of more than one peak in the DOS with different Fermi velocities, the loss function reported in the left column of Figure 13 presents essentially featureless behavior at all q’s. Even though some weak peaks can be discerned in Im$\left[\epsilon^{-1}\right]$ we classify them as single-particle excitations. Hence, in NiH, the electron excitation spectrum in the low-energy domain is dominated by single-particle electron-hole excitations.

A large difference in the low-energy dielectric properties of pure Ni and NiH${}_{x}$ might be helpful in elucidating different mechanisms of relaxation of highly energetic charge carriers in metal nanoparticles forming hybrid plasmonic nanomaterials and, in general, the catalytic activity of plasmonic metals [38]. One of the ways may be the hydrogenization of nickel nanoparticles in existing nickel-based catalysts [39,65,66,67,68,69,70,71,72,73] in an attempt to increase the lifetime of the Ni localized surface plasmon resonance (LSPR). On the other hand, the excitation of SAP in the process of inelastic decay of LSPR might cause stronger confinement of electromagnetic fields at plasmonic catalysts thus enhancing catalytic activities. We believe that Ni nanoparticles might provide a good opportunity to investigate the mechanism of such enhancement.

## 4. Conclusions

In summary, we studied the low-energy collective electronic excitations in Ni and NiH${}_{x}$ in the framework of the time-dependent density-functional theory. In clean Ni, we found several plasmon peaks with strong anisotropy in the calculated loss functions related to the existence of several energy bands with different Fermi velocities. The dispersion of all these peaks presents a sound-like behavior. Since the respective out-of-phase charge oscillations in different spin subsystems also involve variations in the spin structure, we call these modes spin acoustic plasmons (SAPs). At momentum transfers in the [100] direction we find one dominating SAP and two notably weaker ones. In the [110] direction we found two weak SAPs. The excitation spectrum in the [111] direction has two such modes, one of which is dominating.

We varied the hydrogen concentration *x* in PdH${}_{x}$ and traced the evolution of these SAPs. At x=0.25 we observed a strong reduction in the group velocities of SAPs. Moreover, the number of such modes changes as well. This is accompanied by a reduction of the SAP lifetime. We relate such impact of the H absorption on the SAPs to strong modifications in the electronic structure at the Fermi level, and to the reduction of the energy splitting between bands with different spins. After subsequent increase of the H concentration, the number of SAP reduces and its collective character reduces too. Finally, in NiH the electron excitation spectra are entirely dominated by single-particle electron-hole pairs. In view of the discovered strong variations of the low-energy dielectric properties and collective electronic excitations in Ni and NiH${}_{x}$, it appears useful to exploit the hydrogenization of Ni-based catalytic materials in order to elucidate the details of the energy and charge transfers in these technologically important systems.

## Data Availability

Not applicable.

## Abbreviations

The following abbreviations are used in this manuscript:

AP	Acoustic plasmon
SAP	Spin acoustic plasmon
FLAPW	Full-potential linearized augmented plane wave method
BZ	Brillouin zone
LSPR	Localized surface plasmon resonance
